# Current Scenario and Challenges in the Direct Identification of Microorganisms Using MALDI TOF MS

**DOI:** 10.3390/microorganisms9091917

**Published:** 2021-09-09

**Authors:** Sang-Soo Han, Young-Su Jeong, Sun-Kyung Choi

**Affiliations:** 1 Advanced Defense Science & Technology Research Institute, Agency for Defense Development, Daejeon 34186, Korea; sshan0324@add.re.kr; 2 Chem-Bio Technology Center, Agency for Defense Development, Daejeon 34186, Korea; choisk01@add.re.kr

**Keywords:** microbial identification, MALDI TOF MS, machine learning, challenges, application

## Abstract

MALDI TOF MS-based microbial identification significantly lowers the operational costs because of minimal requirements of substrates and reagents for extraction. Therefore, it has been widely used in varied applications such as clinical, food, military, and ecological research. However, the MALDI TOF MS method is laced with many challenges including its limitation of the reference spectrum. This review briefly introduces the background of MALDI TOF MS technology, including sample preparation and workflow. We have primarily discussed the application of MALDI TOF MS in the identification of microorganisms. Furthermore, we have discussed the current trends for bioaerosol detection using MALDI TOF MS and the limitations and challenges involved, and finally the approaches to overcome these challenges.

## 1. Introduction

**Figure 1:**
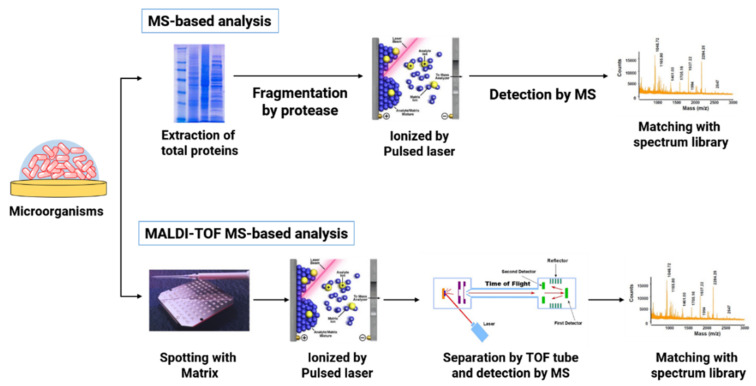
Schematic representation of MS and MALDI TOF MS operation for microorganism identification.

Microbial identification methods that employ biochemical analysis, and 16S or 18S ribosomal RNA (rRNA) gene sequences are expensive and time-consuming [1]. The overall operational cost of a 16S rRNA phylogenetic analysis for each bacterial sample is approximately USD 100 and takes 48 h for completion [2]. The advent of state-of-the-art technologies such as matrix-assisted laser desorption time of flight mass spectrometry (MALDI TOF MS) has ensured faster and reliable results to analyze and identify microorganisms (Figure 1) [3,4,5]. MALDI TOF MS-based microbial identification provides the advantages of a general-purpose sample preparation platform for a plethora of organisms including bacteria, fungi, and yeast, while conventional biochemical analysis requires organism-specific procedures, reagents, and kits. These differences in sample preparation procedures impact the direct and indirect costs of consumables, thus rendering biochemical testing much more expensive [6]. MALDI TOF MS-based identification has significantly lower operational costs, which include the minimum requirements for substrates and reagents for extraction. Additionally, storage costs associated with refrigeration are circumvented.

Abundant information on MALDI TOF MS and its application has been reported in areas of medicine [7], food [6], military science [8], and ecological research [5], and for a broad spectrum of microorganisms ranging from Gram-positive bacteria to Gram-negative bacteria [9], yeasts [10], filamentous fungi [11], protozoa [12], and algae [13]. However, many challenges remain with the MALDI TOF MS method, including the main limitation of the reference spectrum [14]. The mass spectrometric identification of microorganisms relies on identifying the characteristic spectrum of each species, which is compared with the large database in the MS instrument also known as the reference spectrum. Currently, MALDI TOF MS is unsuitable for distinguishing between *Shigella* and *Escherichia coli*, *Bordetella pertussis* and *Achromobacter ruhlandii*, *Achromobacter xylosoxidans* and *Achromobacter ruhlandii*, and *Bacteroides nordii* and *B. salyersiae* [7]. Similarly, the *Enterobacter cloacae* complex, a group of six closely related species (*E. asburiae*, *E. cloacae*, *E. hormaechei*, *E. kobei*, *E. ludwigii*, and *E. nimipressuralis*) possessing similar resistance modes, cannot be distinguished using MALDI TOF MS [15].

This review briefly provides a background on MALDI TOF MS technology, including sample preparation, workflow, and its application in the identification of microorganisms. Furthermore, we have discussed the current trends for bioaerosol detection using MALDI TOF MS and its limitations and challenges.

## 2. MALDI TOF MS-Based Microbial Identification

Samples for MALDI MS analysis are prepared by mixing or coating a solution of an energy-absorbing organic compound called the matrix. When the matrix crystallizes while drying, the sample embedded in the matrix co-crystallizes. The most commonly used matrices for biological samples such as proteins or peptides are α-cyano-4-hydroxynamic acid (CHCA or HCCA), 5-chloro-2-mercaptobenzothiazole (CMBT), sinapinic acid (SA), and dihydroxybenzoic acid (DHB) [16,17,18]. The CHCA is generally used for peptides in the lower mass range (<2.5 kDa) and forms small homogeneous crystals to provide optimal resolution during the MS analysis. The CMBT is usually used to analyze bacterial endotoxins including lipid A with high sensitivity. This matrix supplies the tolerance to high concentrations of reagents such as calcium chloride, sodium chloride and sodium dodecyl sulphate [19]. The SA used to analyze high mass (>2.5 kDa) peptides and proteins also promotes the formation of small crystals. DHB is the preferred matrix for glycoprotein and glycan analysis and is also routinely used in peptide analysis. The advantage of using DHB in peptide analysis is that the matrix is more resistant to contamination (such as from salt and/or detergent) than other matrices.

Desorption and ionization of the sample on the matrix by a laser beam generates individual protonated ions of the analytes in the sample. The protonated ions are then accelerated through a fixed potential and separated from each other according to their mass-charge ratio (*m*/*z*). During MALDI TOF MS analysis, the *m*/*z* ratio of an ion is measured by determining the time it takes for the ion to traverse the length of the flight tube. A few TOF analyzers equipped with an ion mirror at the rear of the flight tube reflect the ions through the flight tube, back to the detector. Therefore, the ion mirror not only increases the length of the flight tube but also corrects for small energy differences between ions. A characteristic spectrum known as the peptide mass fingerprint (PMF) is generated for the analytes in the sample on the basis of the TOF information.

Microbial identification using MALDI TOF MS involves comparing the PMF of the unknown organism with the PMF contained in the database, or by comparing the mass of the biomarker in the unknown organism with the reference database of the proteome [20,21]. In PMF pairing, the MS spectra of unknown microbial isolates are compared to the MS spectra of known microbial isolates in the database [22]. To determine bacterial identity at the species level, a typical *m*/*z* mass range of 2 to 20 kDa is used [23], representing mainly ribosomal proteins as well as some maintenance proteins. The characteristic pattern of ribosomal proteins is abundant, accounting for approximately 60–70% of the dry weight of microbial cells in the mass range of 2–20 kDa and is used to identify a particular organism by modeling its PMF to the PMF of ribosomal proteins [24]. Then, the resulting PMF sample is compared to the spectrum contained in the database according to the specific algorithm software used. Identification occurs after the spectral signature of the proteins is correlated with the spectral database collected from the reference strains. Software and databases are currently commercialized by equipment manufacturers with their systems for routine identification of microorganisms. For example, Bruker (Billerica, MA, USA) MALDI BioTyper, Shimadzu (Kyoto, Japan) SampleStations and AuraSolution, SARAMIS VITEK MS RUO and BioMérieux (Marcy l’étoile, France) Andromas systems offer different types of databases and software. The results are returned to the scoring system. However, the manufacturer-provided MALDI TOF MS database has a successful identification of only 8% of microorganisms in accordance with genetic identification. To overcome this technical bottleneck, researchers created the custom database using a reproducible standard operating process [25]. The scoring system seems conservative enough to prevent a false positive [26]. Thus, the identity of microorganisms can be established to their genus, species, and lineage levels. This approach is widely used in microbial identification owing to its simplicity and convenient application in the diagnostic laboratory, and is supported by the availability of numerous commercial libraries of biological PMFs.

## 3. Applications of MALDI TOF MS Analysis

Microbial identification plays a key role in several areas of research and application. For example, microbes in the food industry are important to accurately identify contaminants leading to spoilage in food. In clinical microbiology, the primary goal is to isolate, identify, and study pathogenic microorganisms. Additionally, a critical military requirement in addition to diagnostic tests is the rapid identification of pathogens, which provides clinicians with vital advantages in bacterial infections, viruses, and other life-threatening biological hazards such as biological weapons.

### 3.1. Clinical Applications

Typically, bacterial infection in body fluids is diagnosed by biochemical and metabolic profiling, requiring 24 to 48 h to identify the bacterial species involved [27]. Meanwhile, the patient is administered empiric, and sometimes inappropriate, antibiotics. Clinical microbiology laboratories require rapid, reliable, and cost-effective methods to identify potential pathogens in clinical samples to initiate an appropriate antibiotic therapy.

The implementation of MALDI TOF MS for the routine identification of microorganisms directly from blood cultures has been shown to significantly impact the rationalization of antibiotics, with a potentially positive effect on the rate of antibiotic resistance [28,29,30]. The clinical impact of performing MALDI TOF MS on blood cultures was evaluated in a study by Clerc et al.; MALDI TOF MS made it possible to adjust the antibiotic treatment in 35.1% of the bacteremia cases analyzed [31]. Excluding the centrifugation steps, the hospital stay was reduced by approximately 2 days, depending on the type of patient and the relevance of the patient’s management. Due to the widespread use of carbapenems for septic shock, the rationalization of antibiotics has been observed more frequently, with the routine application of MALDI TOF MS to blood cultures, thereby having a clear positive effect in reducing the use of carbapenems and other broad-spectrum antibiotics. A recent prospective study confirmed that the identification of the causative agent of sepsis by using MALDI TOF MS resulted in a shorter time frame for adequate antibiotic therapy. In this study, ampC-positive and Gram-negative sepsis patients rapidly identified using MALDI TOF MS received optimal treatment within 48 h [32].

The ongoing emergence of acquired antibiotic/antifungal resistance necessitates results from a full-day antibiotic susceptibility test (AST) [33]. From this perspective, some studies have investigated the use of MALDI TOF MS to perform AST [34,35]. The MALDI TOF AST test was first developed to detect specific peaks of resistant strains, using the peak selection method. However, most of these studies involve the detection of drug hydrolysis/modification. Recently, MALDI TOF MS assays have been designed to detect resistance regardless of biological mechanisms, and to assess the growth of microorganisms in the presence of a given drug [36]. MALDI TOF MS was used to quickly acquire the protein spectrum as a diagnostic tool for identifying infection indicators. Spectra are often collected directly from clinical samples, mostly serum or whole blood, for this purpose. This method is beneficial when pathogens are seldom discovered; this is usually the case for suspected but unproven fungal infections and slow-growing organisms such as *Mycobacteria* species [34].

### 3.2. Food-Microbiological Applications

MALDI TOF MS has been used extensively in food microbiology [6]. Until now, the identification of food-related bacteria based on MALDI TOF MS has focused on food pathogens, such as *Campylobacter* [37], *Listeria* [38], and *Salmonella* [39]. A few reports have evaluated various aspects of the applicability of MALDI TOF to food microbiology, such as the classification of lactic acid bacteria in fermented meat, monitoring of probiotic bacteria in yogurt, and the identification and characterization of bacteria that produce biogenic amines [40].

Recently, the food microbiology laboratory added MALDI TOF MS to its routine microbiological identification process [6]. This technology determines the unique protein fingerprints of microorganisms and is used to reliably identify species, especially by combining their fingerprints with the fingerprints in the entire library. This tool complements existing technologies, such as the use of rDNA sequencing methods for sequence-based identification. This MALDI TOF MS technology enables laboratories to provide flexible response times and cost options to meet customer needs.

### 3.3. Ecological Application

Metagenomics and other culture-independent studies have shown that a diverse population of hundreds of millions of microorganisms thrive in the different ecosystems on Earth [3]. A majority of these strains have not yet been cultured, and their metabolic function is still unknown. The key advantage of microbial cultures is through the isolation of pure cultures for their potential biotechnological applications. Although it is not possible to culture all members of the microbial community, the culture of a majority of them, including several new taxa, is accomplished by mirroring the culture conditions of closely related species.

A few studies have reported the application of MALDI TOF MS to accurately and quickly identify microorganisms isolated from various environments, including hospital environments, biofilm habitats, spacecraft and related surfaces, and mobile phones [26,41,42,43]. However, several studies have concluded that the poor reliability in the identification of microorganisms from non-clinical ecosystems (such as soil, water, and spacecraft assembly cleanrooms) is because of the prevalence of MS profiles of clinical isolates in the database [26,42]. The primary reason for this discrepancy is probably an inadequate range of bacterial species in the database, to which PMF comparisons are usually conducted. The dearth of public repositories for submitting references to new spectra created by researchers exacerbates this problem [21]. Moreover, the absence of specific criteria and tools to verify the accuracy of the reference profile adds to the challenges. However, an attempt is being made to define a general MALDI spectral database.

### 3.4. Military Applications

Since the discovery of biological weapons in Iraq’s arsenal in 1991, research and development of detection technologies for weapons of mass destruction have been strengthened. While 138 people died of anthrax in Sverdlovsk, Russia, 751 people in Oregon were deliberately infected with *Salmonella*. The anthrax vaccine, Black Death, and ricin cultures seized by US military organizations show the importance of efficient detection technologies for the military [44]. A well-equipped fighting force can discover a biological warfare agent (BWA) attack, only 25 to 40 min after it begins. Generally, phenotypic, genotypic, and immunological identification systems have been used to identify organisms that represent a serious threat as vectors of bioterrorism. These systems are slow, cumbersome, and represent a significant risk to laboratory personnel. Recently, many researchers have adopted MALDI TOF MS as a simple, fast, and reliable method to identify highly pathogenic organisms such as *Brucella* spp., *Coxiella burnetti*, *Bacillus anthracis*, and *Francisella tularensis* [45,46,47,48].

**Figure 2:**
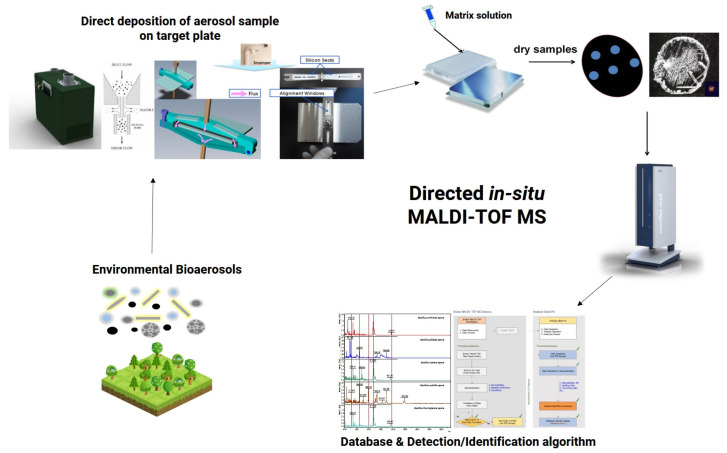
Schematic representation of a direct in situ MALDI TOF MS system for the rapid detection of bioaerosol particles [49,50]. The detection capability of aerosolized BWAs with sizes of 2–10 μm was confirmed by aerosol collection system, which can be most effectively accumulated on respiratory organs. Reprinted with permission from [49]. The Korean Chemical Society.

The spores produce phenotypes very different from the colonies of the BWA bacteria [8]. Jeong et al. reported an in situ direct MALDI TOF MS system enabling the high-throughput detection and identification of aerosolized *Bacillus* spore particles, subsequently building a *Bacillus* spore mass spectrometry database, upon which an algorithm was developed and applied [49]. In addition, bioparticle generation and direct collection systems have been developed to analyze *Bacillus* spore aerosol particles of 2–10 μm, which is the optimal size for BWA. A direct in situ MALDI TOF MS system can rapidly analyze and detect the 2–10 μm *Bacillus* spore aerosol particles without sample pretreatment. To perform real-time detection and identification, a *Bacillus* spore mass spectrometry database was built and algorithms were developed and applied. This approach can be used for the rapid detection and inspection of BWA (Figure 2) [50]. Since organisms used as BWA contain or produce biomolecules responsible for their pathogenic activity, detecting them in mass spectra with reference to BWA standards is an important and growing area of research.

## 4. MALDI TOF MS Analysis for Detection of Bioaerosols

Bioaerosols are biological materials in the air. Bioaerosols can be composed of bacterial cells, toxins, viruses, fungal spores, fungal hyphae, and by-products of the metabolism of microorganisms, which can cause infectious diseases by carrying viruses (e.g., the influenza A H1N1 virus) [51]. Recently, Severe Acute Respiratory Syndrome Coronavirus 2 (SARS-CoV-2) and novel Coronavirus 2019 (COVID-19) has spread globally, posing an unprecedented challenge in recent history to the international public health, education, and trade systems [52]. MALDI TOF MS has been used to detect and characterize viral proteins. However, detecting specific proteins in mass spectra of whole bacteria or extracts has proven to be more difficult. Only a few published studies have recorded the mass spectra of bacterial reference standards and compared them with test samples based on experience [53]. Additionally, BWAs are most effective when deposited in the respiratory tract of humans [44]. Thus, real-time detection of pathogens in bioaerosols can help improve support and management to characterize infected individuals and mitigate the spread of disease.

To identify the cause of infectious diseases through bioaerosol, serological techniques (such as antibody-based enzyme immunoassays) and molecular amplification methods (such as PCR) are generally used because they are more specific, more sensitive, and can be performed faster, although they still require several hours [54]. MALDI TOF MS is being studied as a potential means of detecting bioaerosols. At Lawrence Livermore National Laboratory, a rapid analytical technique called BAMS (bioaerosol mass spectrometry) for sampling and detecting bioaerosols with individual particle-level resolution is used to detect substances in the air [55]. This technique does not require reagents and it reports the mass spectra characteristics of individual spores. Fergenson et al. distinguished single spore particles of *B. thuringiensis* and *B. atrophaeus* based on the presence or absence of a single peak [56]. Kim et al. provided a method to collect and analyze bioaerosols by BAMS using an Andersen N6 bioaerosol collector [57]. The mass spectra obtained by matrix addition after bioaerosol deposition with *E. coli* have an almost better signal-to-noise ratio than those obtained by the dry droplet approach. Stowers et al. reported that mass spectra are generated from aerosols composed of two small biological molecules—gramicidin or erythromycin—or from aerosol spores of *B. atrophaeus* cells [58]. Tobias et al. have shown the application of BAMS to understand the sporulation process of *B. atrophaes* cells [59]. Additionally, Steele et al. have presented a proof-of-concept instrument for the rapid detection of hazardous aerosols in lab and field tests at the San Francisco International Airport [60]. These results suggest that BAMS can provide real-time identification of biological aerosols.

BAMS can detect pathogens and BWAs in real time and automatically, without the need for reagents. This technique does not require sample pretreatment or the addition of a matrix. BAMS shows some success in species-level differentiation, but one of the main disadvantages of BAMS is the inherent hardness of ionization [61]. This characteristic leads to a limited mass range, which is extremely limited when observing bioaerosols, wherein large molecules (such as proteins) will greatly enhance specificity. To increase the mass range and sensitivity of BAMS, Kleefsmans et al. reported a device that can preselect biological aerosol particles from non-biological particles by recording the fluorescence emitted when the particles are irradiated with a laser light of 266 nm [62]. With the current performance of the mass spectrometer, the mass spectrum produced has a high resolution, covering a mass range of up to 85 kDa. The *Erwinia herbicola* mass spectrum shows the ability of an aerosol mass spectrometer to produce high-quality bacterial particle spectra. Russel et al. reported a novel design that utilizes a linear flight tube with delayed extraction and an electrostatic ion guide [63]. This study showed that very high levels of sensitivity were obtained, with 14 zmol (8400 molecules) of gramicidin S detected in a single particle. Czerwieniec et al. showed TOF with SIMION modeling. This modeling effect is amplified at higher *m*/*z* values due to the longer ion flight time [64]. Reflectron TOF analysis permeated less than 2% of ions at *m*/*z* 2000, improved through 28% and 45% ion transmission and linear geometry TOF of the ion-permeable portion and total excision model. The new instrument design revealed improved sensitivity for high masses, as shown when standard particles of cytochrome C (*m*/*z* ~12,000) were used. From these particles, monomers, dimers (*m*/*z* ~24,000) and trimers (*m*/*z* ~36,000) of compounds can be obtained. These results show that the MALDI TOF MS analysis demonstrates the potential of protein detection in bioaerosols. However, it also has the problem of limited sensitivity. Future instruments will require the use of BAMS for efficient detection of high mass ions and new ionization technology provides the opportunity to produce higher mass ions. The study of these new possibilities will be an important point for MALDI TOF MS to address its limitations.

## 5. Emerging Technologies to Overcome Limitations of the MALDI TOF MS Analysis

A traditional MALDI TOF MS analysis has several limitations. First, comparing the PMF of an unknown isolate with the reference quality fingerprints present in the database is the most critical step for species identification. It requires a database that not only contains the reference quality fingerprints of all species of interest but also encompasses multiple strains of each fingerprint species. Second, considerable biomass is required to obtain reliable identification results. Although some authors suggest a limit of detection at 6 × 10^3^ CFU/spot, in practice, a limit of 1 × 10^5^ CFU/spot is often required [9,65]. These limitations of the MALDI TOF MS will need to be addressed in the future.

Recently, to address the limitations of MALDI TOF MS, traditional methods such as biochemical testing, serotyping, and genetic analysis have been used to support identification. Many studies have shown that the fingerprint region (900–1200 cm^−1^) of bacterial polysaccharides in Fourier transform infrared spectroscopy (FTIR) exhibits genetic polymorphism and chemical heterogeneity at the species and serotype levels [66]. Feng et al. reported that FTIR can be used to complement the MALDI TOF MS for the identification and typing of taxonomic microorganisms [67]. In their study, 14 strains of *E. coli* and nine strains of *Shigella* were identified using MALDI TOF MS and FTIR techniques. In addition, a data-aggregation strategy using these two approaches was attempted to improve the typing precision. Hierarchical clustering analysis (HCA) showed that the typographic accuracy for *E. coli* and *Shigella* selected from blood agar was 100% for MALDI TOF MS, when combined with FTIR. Additionally, Clark et al. designed a MALDI TOF mass analysis data collection and bioinformatics pipeline (IDBac) to integrate complete protein and specialty metabolite spectral data directly from bacterial cells grown on agar [3]. This technique allows for the comparison of bacteria composed of very similar phylogenetic groups, and to compare metabolic differences in hundreds of isolates within a few hours.

Machine learning (ML) methods can identify statistical dependencies in data while considering the nonlinearity and interaction effects between features [68]. Following current advances, machine learning technology can unravel novel information embedded in the MALDI TOF mass spectrum [69]. This information is useful for the identification and differentiation of species, especially those that are phylogenetically closer at the subspecies level. Van Oosten et al. demonstrated an application of this proof-of-concept in the screening of antibacterial drugs acting on major target proteins such as ribosomes, penicillin-binding proteins, and topoisomerases, in a pharmacologically relevant phenotypic environment, by combining MS and ML [70]. In addition, while 27 studies employed ML for species identification, nine studies used ML for antimicrobial susceptibility testing [71]. Papagiannopoulou et al. demonstrated that single-cell MALDI TOF MS data can be used to identify pathogenic bacteria in urine samples [72]. Although the rapid reaction time (in minutes or seconds) of single-cell MALDI TOF MS is a clear improvement over traditional MALDI TOF MS, it poses additional problems associated with changes in the mass spectrum. Hence, they combined a single-cell MALDI TOF MS with an ML algorithm to experimentally demonstrate that the resulting spectra were useful in distinguishing between different bacterial species.

## 6. Conclusions and Future Directions

MALDI TOF MS is widely used for the routine identification of microbial pathogens, and is replacing existing identification methods, including biochemical and 16S or 18S rRNA gene sequencing, thereby impacting clinical diagnosis [73]. New perspectives for MALDI TOF MS in microbial identification are being explored, such as in the analysis of direct-positive blood cultures to identify pathogens, subspecies and strains, the detection of drug resistance determinants, and the generation of MS spectra of specialized metabolites to evaluate the functional characteristics of bacteria. MALDI TOF MS applications are in the early stages of development, laced with challenges, and require further standardization to provide reliable solutions. A combinatorial approach with other methods such as FTIR and ML algorithms, and the development of new sample preparation methods has improved the application of MALDI TOF MS, increasing its relevance in microbial studies. Further standardization and convenient access to MS databases from various sources, not solely clinical sources, will render it the preferred method for microbial research and industrial applications. Additionally, the directed in situ MALDI TOF MS technology will be useful in the real-time detection and identification of disseminated biological warfare agents such as *B. anthracis* spores as well as other microorganisms without requiring tandem MS, extraction steps and mechanical disruption methods in the field. The application of MALDI TOF MS in high-throughput screening and identification studies is increasing rapidly with the advances in automation.
